# Correction: WIPO initiative of transforming traditional resources and sharing rights: An evolutionary game analysis and a Chinese context

**DOI:** 10.1371/journal.pone.0314303

**Published:** 2024-11-18

**Authors:** Dong Zhang, Rui Huang, Chaoran Lin

The Funding statement for this paper is incorrect. The correct Funding statement is as follows: This work was supported by the Ministry of Education of the People’s Republic of China (grant number 23YJAZH191, http://www.moe.gov.cn/s78/A13/tongzhi/202310/t20231019_1086367.html) for the project titled “Research on the Path of Traditional Resource Rights Sharing from the Perspective of Creative Transformation,” awarded to DZ. The funders had no role in study design, data collection and analysis, decision to publish, or preparation of the manuscript.
