# Supplementary material for: WIPO initiative of transforming traditional resources and sharing rights: An evolutionary game analysis and a Chinese context
Source: PLoS One. 2024 May 21;19(5):e0297885. doi: 10.1371/journal.pone.0297885 (PMC11108203; doi:10.1371/journal.pone.0297885)
Supplement: Attachment — Submitted filename: Review_Comments.pdf [file pone.0297885.s001.pdf]

Dear Authors,

As the negotiations for WIPO-GRTKF progress, the international system for sharing rights arising from the transformation of traditional resources is being restructured.

This study has presented an empirical model on how holders and users can collaborate to transform traditional resources and share rights. The proposed model asserts that international negotiators can diversify rights and make more empirical judgments about the process of rights-sharing. Thus, in this paper, the rights arising from transforming and utilizing traditional resources are organized from the following four perspectives: 1. A Robust Structure: Valuing Respect and Sharing. 2. Stakeholder Game Relations: Holders and Users 3. Components of Subject: Holders and Users 4. Game Relationships: An Up-Close View.

Based on the four perspectives, this paper applies the "Evolutionary Game Reasoning and Analysis" methodology that embodies these perspectives and validates situations for managing rights arising from resource transformations through formulas (Eq.1~Eq.5). Through game simulation, it is shown that while the access approach to rights sharing may be a necessary condition when it comes to the transformation of traditional resources, it is not a sufficient condition, since users and holders can make strategic choices (Figure 2, Page 10). In other words, in this paper, the authors explain the sharing and management of rights arising from the transformation of traditional resources based on an empirical model. However, evaluating the proposed empirical model more objectively and quantitatively seems necessary. For example, it is necessary to show clear evidence supporting the paper's claims by presenting experimental results of the empirical model, possibly by applying more cases than those mentioned in Table 2, or by diversifying the factors considered.

Additionally, it is essential to provide a detailed introduction to the data (instances) used in the proposed empirical model. Access pathways to the datasets should be available for readers to replicate the study.

Finally, on Page 7, there is a need to consistently edit the indentation of the first bracketed number in each paragraph.
