# Supplementary material for: WIPO initiative of transforming traditional resources and sharing rights: An evolutionary game analysis and a Chinese context
Source: PLoS One. 2024 May 21;19(5):e0297885. doi: 10.1371/journal.pone.0297885 (PMC11108203; doi:10.1371/journal.pone.0297885)
Supplement: Attachment — Submitted filename: Response to Reviewer.docx [file pone.0297885.s002.docx]

Response to Reviewers

**PONE-D-23-24767**

**WIPO Initiative of Transforming Traditional Resources and Sharing Rights: An Evolutionary Game Analysis and A Chinese Context**

The authors appreciate the opportunity to submit a revised draft of the manuscript “WIPO Initiative of Transforming Traditional Resources and Sharing Rights: An Evolutionary Game Analysis and A Chinese Context” for publication in PLOS ONE. We appreciate the time and effort that the academic editor and the two reviewers dedicated to providing comments and suggestions, which have helped us implement valuable improvements to our paper. We have addressed all the journal requirements and incorporated most of the suggestions made by the reviewers in the revised paper, detailed below in point-by-point responses. We have attached the revised paper with changes highlighted in addition to a “clean” copy. We will be willing to make further revisions if necessary.

Journal Requirements:

1. Please ensure that your manuscript meets PLOS ONE's style requirements, including those for file naming. The PLOS ONE style templates can be found at

https://journals.plos.org/plosone/s/file?id=wjVg/PLOSOne_formatting_sample_main_body.pdf and

<https://journals.plos.org/plosone/s/file?id=ba62/PLOSOne_formatting_sample_title_authors_affiliations.pdf>

Author response:

We have formatted the revised paper following PLOS ONE style requirements, using the PLOS ONE style templates.

2. In your Data Availability statement, you have not specified where the minimal data set underlying the results described in your manuscript can be found. PLOS defines a study's minimal data set as the underlying data used to reach the conclusions drawn in the manuscript and any additional data required to replicate the reported study findings in their entirety. All PLOS journals require that the minimal data set be made fully available. For more information about our data policy, please see http://journals.plos.org/plosone/s/data-availability.

""Upon re-submitting your revised manuscript, please upload your study’s minimal underlying data set as either Supporting Information files or to a stable, public repository and include the relevant URLs, DOIs, or accession numbers within your revised cover letter. For a list of acceptable repositories, please see http://journals.plos.org/plosone/s/data-availability#loc-recommended-repositories. Any potentially identifying patient information must be fully anonymized.

Important: If there are ethical or legal restrictions to sharing your data publicly, please explain these restrictions in detail. Please see our guidelines for more information on what we consider unacceptable restrictions to publicly sharing data: http://journals.plos.org/plosone/s/data-availability#loc-unacceptable-data-access-restrictions. Note that it is not acceptable for the authors to be the sole named individuals responsible for ensuring data access.

We will update your Data Availability statement to reflect the information you provide in your cover letter.

Author response:

Below is our revised Data Availability statement.

We have made the Matlab running code for the simulation of the evolutionary game results available in our GitHub project. This will allow others to access the same data and simulation code analysed in this study. These data can be viewed on the GitHub database website via the link below:

<https://github.com/huangruiipr/Traditional-Resource-Evolution-Game.git>

Data can also be requested from the corresponding author upon reasonable request.

3. Please include your full ethics statement in the ‘Methods’ section of your manuscript file. In your statement, please include the full name of the IRB or ethics committee who approved or waived your study, as well as whether or not you obtained informed written or verbal consent. If consent was waived for your study, please include this information in your statement as well.

Author response:

Regarding the request for a full ethics statement in the 'Methods' section of our manuscript, I would like to clarify that our study did not involve any activities that would require ethical review and approval.

Our research did not involve human participants or animals, and no personal or sensitive data was used. Therefore, it was not necessary to seek approval from an Institutional Review Board (IRB) or ethics committee.

However, we understand the importance of ethics in research and assure you that our study was conducted in accordance with the general ethical guidelines of our field.

We have updated the 'Methods' section of our manuscript to clearly state this information and to avoid any potential confusion for readers.

Thank you again for bringing this to our attention. We appreciate your efforts in helping us improve our manuscript.

Reviewers' comments:

Reviewer's Responses to Questions

**Comments to the Author**

1. Is the manuscript technically sound, and do the data support the conclusions?

The manuscript must describe a technically sound piece of scientific research with data that supports the conclusions. Experiments must have been conducted rigorously, with appropriate controls, replication, and sample sizes. The conclusions must be drawn appropriately based on the data presented.

Reviewer #1: Partly

Reviewer #2: Partly

2. Has the statistical analysis been performed appropriately and rigorously?

Reviewer #1: I Don't Know

Reviewer #2: No

3. Have the authors made all data underlying the findings in their manuscript fully available?

The [PLOS Data policy](http://www.plosone.org/static/policies.action#sharing) requires authors to make all data underlying the findings described in their manuscript fully available without restriction, with rare exception (please refer to the Data Availability Statement in the manuscript PDF file). The data should be provided as part of the manuscript or its supporting information, or deposited to a public repository. For example, in addition to summary statistics, the data points behind means, medians and variance measures should be available. If there are restrictions on publicly sharing data—e.g. participant privacy or use of data from a third party—those must be specified.

Reviewer #1: No

Reviewer #2: No

4. Is the manuscript presented in an intelligible fashion and written in standard English?

PLOS ONE does not copyedit accepted manuscripts, so the language in submitted articles must be clear, correct, and unambiguous. Any typographical or grammatical errors should be corrected at revision, so please note any specific errors here.

Reviewer #1: Yes

Reviewer #2: No

5. Review Comments to the Author

Please use the space provided to explain your answers to the questions above. You may also include additional comments for the author, including concerns about dual publication, research ethics, or publication ethics. (Please upload your review as an attachment if it exceeds 20,000 characters)

Reviewer #1: As the WIPO-GRTKF negotiations unfold, the international framework for sharing rights, stemming from the transformation of traditional resources, is undergoing revision. This study introduces an empirical model detailing how resource holders and users might collaborate to adapt traditional resources and share associated rights. The model suggests that international negotiators can diversify rights and base their decisions on empirical evidence regarding the rights-sharing process.

In this manuscript, the authors categorize the rights related to the transformation and utilization of traditional resources under four main perspectives:

A Robust Structure: Valuing Respect and Sharing
Stakeholder Game Relations: Holders vs. Users
Components of Subject: Differentiating Holders from Users
Game Relationships: A Detailed Examination
Moreover, encapsulating these perspectives, the "Evolutionary Game Reasoning and Analysis" methodology is employed. The authors validate scenarios for managing the rights originating from resource transformations using specified equations (Eq.1~Eq.5).

The game simulation indicates that the access approach to rights sharing, while necessary for traditional resource transformation, doesn't suffice. This is because both users and holders possess the autonomy to make strategic decisions (as depicted in Figure 2, Page 10).

Author response:

We greatly appreciate the reviewer’s recognition of the strengths and potentials of our study.

To put it briefly, this manuscript offers insights into rights sharing and management derived from the transformation of traditional resources, all grounded in an empirical model. Nonetheless, a more objective and quantitative assessment of the proposed model appears warranted. The authors might consider furnishing the empirical model's experimental results to bolster the assertions. This could involve broadening the scope beyond the instances in Table 2 and integrating a more diverse set of factors.

Author response:

In the second part of the article, We have carried out a more objective quantitative assessment of the model. In the third section of the second part of the article, We make systematic improvements and additions to the simulation and validation of the model. We highlight the rationale for not requiring a database for the evolutionary game model on page 9 of the article. In the simulation section we have considered and analysed all the factors affecting the conversion and sharing of traditional resource rights and interests, and we have explained the criteria and sources for choosing the values (see page 9, para 1). At the same time, the graphs produced in this study were optimised. We redrew the diagram of the evolutionary game process of traditional resource rights conversion and sharing(see Fig 2), and added the analysis of the influence of different factors on the results of the evolutionary game (see page 9,10,11) .

Among them，Figure 3 reflects the impact of traditional resource equity transformation and sharing of total gain on the system evolution results, the graph is the simulation results of $x$ variable with two obvious differentiation options with the change of $R_{1}$ , and $y$ variable with the change of $R_{1}$ from agreeing strategy to disagreeing strategy (see page 9). Figure 4 reflects the effect of third-party funding on the evolutionary outcomes of the system, with the graphs showing the simulation results for the $x$ variable with two distinct divergent choices as $R_{4}$changes, and the $y$variable with $R_{4}$as it changes from an agreeing strategy to a disagreeing strategy (see page 10). Figures 5, 6 and 7 give the results of the system evolution and the effect on the system evolution rate as application fee，skill transfer cost and database building expense change, respectively (see page 10).

Furthermore, the formatting on Page 7 requires attention, particularly the consistent indentation of the first bracketed number in each section.

Author response:

Thank you to the reviewers for their thoughtful suggestions. We have corrected the formatting where it was incorrect and this has resulted in consistent indentation of the numbers in brackets in each section.

Reviewer #2: abstract is too wordy. make it simple to the readers

Author response:

Abstracts of articles have been streamlined. The revisions have been highlighted in the revised paper. In particular, the background to the writing of the article and the use of evolutionary gaming methods are presented more succinctly.

no references for the statements in the introduction

Author response:

We have added references in the introduction. Reference I is a new addition.

can include more related latest references

references are not well arranged. It shall be arranged as per the format given

Ensure all the reference are cited.

Author response:

We have cited the latest references and organised the references in the format given. Revised the labelling in the article where references were not added. In particular, the formatting of references 2, 15, 22 and 29 has been corrected.

when it is related with chinese context, then why the authors want to take the cases in india

Author response:

In the third part of the article, examples are also given to illustrate relevant cases of conservation of traditional resources in Australia. China context exploration need a comparative study with other countries which also being rich in traditional resources. For this purpose, authors quote some representative cases not only Divya Pharmacy v. Union from India but also the case Aboriginal artworks from Australia.

Results and Discussion missing, some comparison with the proposed approach need to be added

Author response:

We have added Results and Discussion. It focuses on the comparison of the stability of evolutionary game models under different selection strategies, with an emphasis on the key factors affecting the stability of the model, and how the probability of strategy selection by holders and users affects the outcome of the transformation of traditional resource interests.

The flow of the paper should be improved.

The language in the paper should be improved.

Author response:

Many thanks to the reviewers for their suggestions on the logic and language of the article. We have improved the flow of the article and endeavoured to improve the English presentation. The sections and structure of the article have been changed in accordance with the requirements of the journal and are structured as Introduction, Materials and Methods, Results, Discussion and Conclusion. The first chapter was shortened to give more emphasis to the innovation of an evolutionary game analysis of the conversion process of traditional resource rights and interests.

Conclusion should clearly state the outcome.

Author response:

We modified the presentation of the paper's conclusion to make it a more direct statement of the results. In the conclusion, the role of the evolutionary game model for the transformation and sharing of traditional resource rights is directly stated against the "Results and Discussion" in Part III of the paper. When transforming traditional resources, to increase the probability of users and holders choosing a rights-sharing model，the parties need to increase the total benefits from the conversion and utilisation of traditional resources , seek third-party financial support(see page 17).

6. PLOS authors have the option to publish the peer review history of their article ([what does this mean?](https://journals.plos.org/plosone/s/editorial-and-peer-review-process#loc-peer-review-history)). If published, this will include your full peer review and any attached files.


If you choose “no”, your identity will remain anonymous but your review may still be made public.


**Do you want your identity to be public for this peer review?** For information about this choice, including consent withdrawal, please see our [Privacy Policy](https://www.plos.org/privacy-policy).

Reviewer #1: No

Reviewer #2: No
